# Predicting Extended-Spectrum Beta-Lactamase and Carbapenem Resistance in Enterobacteriaceae Bacteremia: A Diagnostic Model Systematic Review and Meta-Analysis

**DOI:** 10.3390/antibiotics12091452

**Published:** 2023-09-17

**Authors:** Tristan T. Timbrook, McKenna J. Fowler

**Affiliations:** 1 Department of Pharmacotherapy, University of Utah College of Pharmacy, Salt Lake City, UT 84112, USA; mckenna.fowler@pharm.utah.edu; 2 BioMérieux, 69280 Marcy l’Etoile, France

**Keywords:** Enterobacteriaceae bacteremia, antimicrobial resistance, ESBL production, carbapenemase production, risk prediction scoring systems, clinical utility of predictive models, antimicrobial stewardship

## Abstract

Enterobacteriaceae bacteremia, particularly when associated with antimicrobial resistance, can result in increased mortality, emphasizing the need for timely effective therapy. Clinical risk prediction models are promising tools, stratifying patients based on their risk of resistance due to ESBL and carbapenemase-producing Enterobacteriaceae in bloodstream infections (BSIs) and, thereby, improving therapeutic decisions. This systematic review and meta-analysis synthesized the literature on the performance of these models. Searches of PubMed and EMBASE led to the identification of 10 relevant studies with 6106 unique patient encounters. Nine studies concerned ESBL prediction, and one focused on the prediction of carbapenemases. For the two ESBL model derivation studies, the discrimination performance showed sensitivities of 53–85% and specificities of 93–95%. Among the four ESBL model derivation and validation studies, the sensitivities were 43–88%, and the specificities were 77–99%. The sensitivity and specificity for the subsequent external validation studies were 7–37% and 88–96%, respectively. For the three external validation studies, only two models were evaluated across multiple studies, with a pooled AUROC of 65–71%, with one study omitting the sensitivity/specificity. Only two studies measured clinical utility through hypothetical therapy assessments. Given the limited evidence on their interventional application, it would be beneficial to further assess these or future models, to better understand their clinical utility and ensure their safe and impactful implementation.

## 1. Introduction

More than 2.8 million antibiotic-resistant infections occur in the United States each year, and more than 35,000 people die as a result [1,2]. Resistant pathogens of major global health concern on the CDC’s urgent and serious threat lists include carbapenem-resistant Enterobacteriaceae (CRE) and extended-spectrum-beta-lactamase-producing Enterobacteriaceae (ESBL-E) [1]. The use of broad-spectrum antibiotics has resulted in the development of resistance in many bacterial pathogens [3]. Thus, the use of broad-spectrum agents, such as carbapenems, is often avoided when possible [4]. This conundrum of ineffective antimicrobial medications and resistant microorganisms places a burden on patients and healthcare providers [5]. Identifying the risk factors for resistant Gram-negative infections is imperative in guiding appropriate treatment interventions and reducing the utilization of broad-spectrum antibiotics [6]. Moreover, employing these initiatives to accurately predict antimicrobial resistance and assign targeted effective treatment is crucial to the recovery of patients with resistant Gram-negative bloodstream infections (GN-BSIs) [7].

Currently, published models exist that assess clinical risk factors for predicting whether a patient has a resistant Gram-negative bloodstream infection [8]. These models often assess factors such as hospitalization, previous colonization, previous antibiotic therapy, age, catheter use, indwelling hardware, surgery, etc. [6]. The application of prediction models can support antimicrobial stewardship efforts by aiding the selection of appropriate treatments and reducing the selection pressure from antibiotics that leads to resistant pathogens [6]. The prediction of resistance in these organisms can be enhanced through evaluation of the performance of published prediction models. This could enhance the understanding of the clinical performance of these models, and their utility in healthcare institutions for effective clinical decision-making. A review of the risk factors and scoring systems used to predict ESBL-E and CRE infections was recently published [8]. However, systematic evaluations and the potential pooled performance of such tools are lacking.

Given the increasing threat from antibiotic-resistant infections, and the gaps in the literature regarding the prediction of ESBL-E and CRE bloodstream infections, this diagnostic model systematic review aims to evaluate the performance of the available models in the literature. This could inform healthcare institutions’ decision-making processes via evaluation of the integration of such models into their clinical procedures to reduce healthcare costs and improve patient outcomes. Moreover, this study could facilitate the adoption of high-performing models to further enhance effective clinical decision-making and antimicrobial stewardship efforts. This diagnostic model systematic review aims to synthesize the available literature to evaluate the performance and discriminatory capabilities of the published models used in predicting ESBL and carbapenemase-producing Enterobacteriaceae bloodstream infections.

## 2. Results

### 2.1. Study Selection

**Figure 1:**
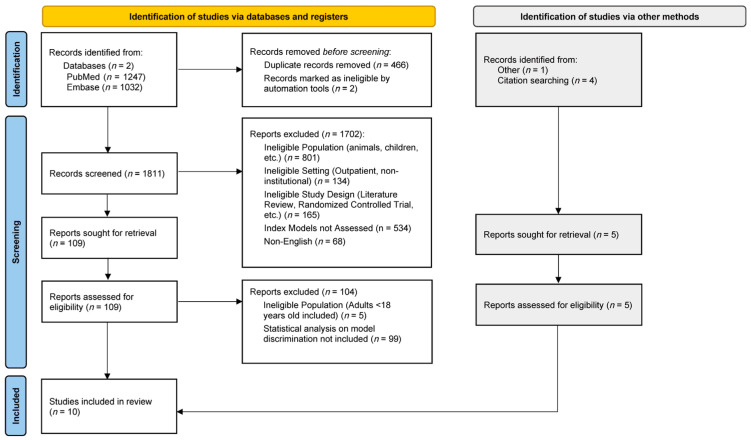
The study selection procedure.

The literature search resulted in 2279 studies that met our keyword query (Figure 1). After the removal of duplicates, we searched 1811 studies, and this resulted in five studies identified for inclusion. Searching the bibliographies of the included studies identified an additional four studies for inclusion. One additional study was included from a conference proceeding. In total, data were extracted from 10 studies for analysis, with 6106 unique patient encounters [9,10,11,12,13,14,15,16,17,18].

### 2.2. Study and Model Characteristics

The characteristics of the studies included can be found in Table 1. The studies were primarily conducted in the United States (70%). Nearly all the studies had a retrospective cohort design (80%), with two retrospective case–control designs, and no prospective studies. The sample sizes varied, ranging from 145 to 1288 patients. The majority of studies (90%) were ESBL models with a resistance incidence ranging from 5.7% to 33.3%, and a median of 12.7%. A single study was of a CRE model, and did not report resistance incidence, as it was a case–control study. Among the 10 studies, 19 analyses of models were evaluated, with four (21.1%) being derivation-based, five (26.3%) being derivation- and internal-validation-based, and ten (52.6%) being external-validation-based.

The demographic details across the studies were heterogeneously reported. Patients predominantly had a median or mean age in the mid-60s, with the female representation ranging from 45.4% to 56.9% (Supplementary Table S1). The racial and ethnic distributions demonstrated substantial diversity, with African American individuals constituting up to 72.5% of patients in some cohorts, while, in others, White individuals comprised up to 47.2%, though the majority of the studies did not report race or ethnicity. Diabetes was consistently highlighted as a significant comorbidity, with prevalence rates as high as 40.1%. Other notable comorbidities included congestive heart failure (up to 50.2%) and end-stage renal disease (23.9%). The source of infection was consistently highest for urinary tract infections, ranging from 31.7% to 58.6%. Although the organisms evaluated varied across studies, *E. coli* was the dominant causative organism, observed in proportions ranging from 56% to 77.2%, followed by *K. pneumoniae*, with figures fluctuating between 13.5% and 40%, except for the single carbapenem-resistance-prediction study, which focused solely on *K. pneumoniae*.

Though the models displayed variations in the risk factors for resistance, certain factors consistently emerged across studies (Table 2). Regarding demographics, age was a predictor in two studies. Hospitalization or admission histories were also common predictors, including prior hospitalizations, recent emergency department visits, and having received care in high-burden regions or abroad. A variety of medical histories were frequently used as predictors, including ESBL-E infection or colonization, in addition to comorbidities or medical exposures, such as COPD, emphysema, ventilator dependence, and urological diseases, and the presence of chronic indwelling hardware. Procedures or interventions were predictors in two studies, and included invasive procedures and GI/GU procedures. Medication-use history was a consistent predictor with antibiotic exposure, both in terms of the duration (days or weeks), and with specific mentions of antibiotic classes, such as β-lactams and fluoroquinolones.

### 2.3. Results of Model Performance in Individual and Pooled Studies

**Figure 2:**
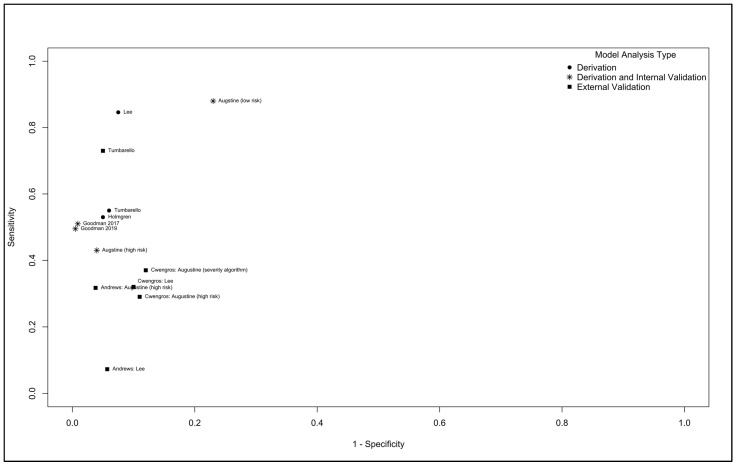
The discrimination performance of ESBL prediction models studies.

**Figure 3:**
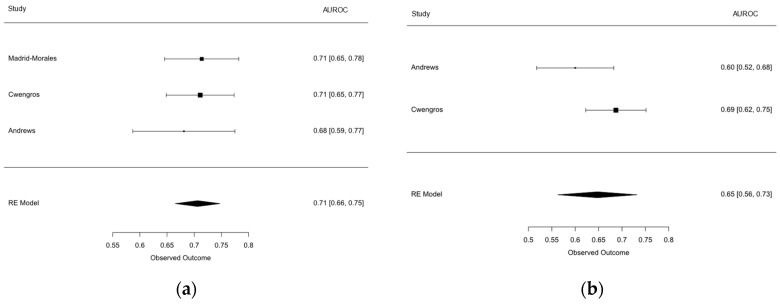
The pooled discrimination performance of external validations. (**a**) Augustine 2017 [10] ESBL model. (**b**) Lee 2017 [15] ESBL model.

Where reported, the discrimination performance based on the sensitivity and specificity of derivation, internal validation, and external validation studies for ESBL models can be found in Figure 2. The derivation performance ranged in sensitivity from 53 to 85%, and in specificity from 93 to 95%, with medians of 55% and 94%, respectively. The derivation and internal validation performance ranged from 43 to 88% for sensitivity and 77 to 99% for specificity, with medians of 43% and 90%, respectively. With the exception a single study that performed derivation and an external validation (Tumbarello; external validation sensitivity 73%, and specificity 95%), subsequent studies performing external validations had a performance that ranged from 7 to 37% for sensitivity, and from 88 to 96% for specificity, with medians of 32% and 90%, respectively. One external validation study did not report the sensitivity and specificity performances, but did report the AUROCs for the Augustine, Goodman 2016, Goodman 2019, and Tumbarello models, which were 0.71 (95% CI 0.65–0.78), 0.59 (95% CI 0.52–0.66), 0.74 (95% CI 0.67–0.81), and 0.76 (95% CI 0.69–0.82), respectively. Two models were externally evaluated in more than one study, and had a pooled discrimination performance, with AUROCs of 0.71 (95% CI 0.66 to 0.75) and 0.65 (95% CI 0.56 to 0.73) (Figure 3). Only three studies reported calibration evaluations, all reporting an adequate fit either from calibration plots, Hosmer–Lemeshow testing, or both. No studies published their model equations.

### 2.4. Clinical Utility

Only two studies (20%) evaluated the potential therapy impacts of model use. In Cwengros et al., using the Lee model, 10% were overtreated with ceftriaxone-susceptible isolates, and 69% were undertreated for resistant ones. Using a severity-of-illness-stratified Augustine model, Cwengros et al. reflected 12% overtreated, and 63% undertreated. Finally, using the Augustine model without severity-of-illness stratification, Cwengros et al. showed results of 11% overtreated and 71% undertreated. Similarly, Andrews et al. observed that 6% of patients with susceptible isolates were overtreated via the Lee model, and that 93% of resistant isolates were undertreated. Using the Augustine model without severity-of-illness stratification, Andrews et al. noted results of 4% overtreated and 68% undertreated. Both the study by Cwengros and that by Andrews et al. evaluated the total carbapenem consumption per 1000 patient days, with similar results.

## 3. Discussion

Due to the increasing burden of antimicrobial resistant infections, tools are needed to improve appropriate antimicrobial use, and achieve the antimicrobial stewardship goals of providing the right drug to the right patient at the right time. In our systematic review of the literature, we found 10 studies with 19 analyses of various models for ESBL and carbapenem resistance among Enterobacteriaceae bloodstream infections. While ESBL derivation studies noted a moderate performance, with the median sensitivity and specificity of 55% and 94%, subsequent studies with external validations reflected a lower median sensitivity and specificity of 32% and 90%, respectively. Two ESBL models with multiple external validations showed a pooled performance with AUROCs of 65% and 71%. Clinical utility evaluations were limited to two studies that reflected a low overtreatment in ceftriaxone-susceptible isolates with the use of models, but a substantial (>60%) undertreatment in ceftriaxone-resistant ones. Given these findings, while some models may limit overtreatment, there is still a critical need to refine and improve current models, to address the substantial risk of undertreatment, ensuring optimal patient care, and advancing antimicrobial stewardship goals.

Endorsed by Infectious Diseases Society of America guidelines, the local validation of risk prediction models, and thorough reporting, are paramount to understanding their performance and potential clinical utility [19]. In our review, less than half of the studies were external validations. Notably, where these validations were performed, they demonstrated an inferior performance compared to the original studies—a decrease which is often seen when models undergo external validation [20].

Our understanding of a model’s generalizability and reproducibility is facilitated by external validation [21]. Specifically, this process can clarify the model’s discrimination (the ability to correctly classify patients into risk groups) and calibration (the alignment between the observed vs. predicted risks across predicted risk ranges) [22]. A significant limitation we observed was that none of the derivation models disclosed their equations, impeding their use for external calibration assessments by frontline clinicians. These studies opted to transform the equations into risk scores for easier external application. Yet, this omission also prohibits the often necessary re-calibration of the risk model’s intercept for differences in baseline risk, particularly when considering the observed heterogeneity in ESBL rates. Given such variations in baseline risk and predictor performance, the external application of models will generally require a model rebuild, rather than reliance on risk scores.

Only three of the studies we analyzed reported calibration performance evaluations, a key metric that is crucial to understanding how prediction models perform across diverse risk levels. The nuances of the demographics and health backgrounds in these studies present a significant variable. For instance, despite differing demographics, such as the elderly veteran population, in the Madrid-Morales study, its discrimination performance was unexpectedly comparable to the Cwengros study. Such similarities in discrimination, despite potential age-related susceptibilities in predicting resistance or varying comorbidities such as COPD, suggest that, while the discrimination might align, the calibration could vary significantly, due to differences in the distribution of predictors across studies. In essence, two models might classify risk similarly (discrimination), but could differ in how accurately those risk predictions match the actual outcomes (calibration) when applied to different populations. This underlines the critical importance of calibration evaluations, which provide insight into how well predictions align with actual outcomes across different risk strata and varied predictor distributions. Given these observations, the comprehensive reporting of calibration evaluations becomes paramount for ensuring the robust application of prediction models across diverse settings and populations.

In our review, only two of the reviewed studies reported methods of sample-size consideration for their prediction model, and these indicated a planned adherence to historical minimal recommendations of 10 events per variable (EPV) for modeling sample size requirements [23]. Surprisingly, no study undertook formal sample-size calculations for prediction modeling, and one study did not meet the minimal EPV recommendations. Given the elements noted above regarding the overall methodological approach of current studies, we strongly advocate for future studies to adhere to the Transparent Reporting of a Multivariable Prediction Model for Individual Prognosis or Diagnosis (TRIPOD) Reporting Guidelines. Additionally, it is imperative that future studies employ the available tools for sample size evaluations, and consistently evaluate and report the calibration performance of their models [24,25,26].

Clinical utility evaluations of predictive models are important in understanding the utility of a model beyond its discrimination and calibration performance; however, few studies in our review evaluated clinical utility [27]. As noted, in the two studies evaluating clinical utility, only the hypothetical undertreatment, overtreatment, and carbapenem use per 1000 patient days was evaluated. However, the collaborators of the Augustine study recently published a separate subsequent study evaluating the implementation of their risk prediction scoring on patient management [28]. For their implementation, they made the prediction score and algorithm available in print, on their internal website for providers, and through a mobile app. They noted a significant decrease in the time taken to administer appropriate antimicrobial therapy from 78 h to 46 h after the implementation of the risk prediction score (*p* = 0.04). This study offers a promising testament to the potential of locally derived, internally validated, and clinically implemented ESBL bloodstream infection risk scoring tools.

However, the landscape of resistance prediction is not confined to these tools alone. Of particular note, genotypic prediction of resistance from diagnostic testing has been shown to outperform some ESBL risk-prediction models [11]. There are myriad factors to weigh when selecting a tool for clinical utility and local validation, as delineated elsewhere [8]. In light of such advancements, institutions should remain adaptive, continuously evaluating a broad spectrum of tools. These evaluations should align with their unique patient demographics and institutional needs, and the evolving epidemiology of resistance [29,30].

An additional important consideration in our review is the inclusion of only a single study focusing on predicting carbapenem resistance in Enterobacteriaceae bloodstream infections. The limited representation of CRE is noteworthy for several reasons. Firstly, CRE is an extremely clinically challenging infection to manage, associated with significant morbidity and mortality [31]. Secondly, CRE detections in bloodstream infections are much more infrequent in the United States, where the majority (70%) of the models included in our systematic review were developed, possibly reflecting differences in clinical practice and research priorities versus countries with higher detection rates. Finally, the singular representation of CRE in our results may reflect a publication bias or a gap in the existing research that warrants future studies. However, despite its limited representation, the inclusion of the CRE prediction model in Enterobacteriaceae bloodstream infections serves to highlight the current state of the literature on prediction model tools to support patient management and antimicrobial stewardship, along with the need for additional research in this area.

There are several limitations to our review. The selection of studies was limited to studies evaluating risk modeling for ESBL and carbapenem resistance in Enterobacteriaceae in the bloodstream, which may limit practical applications, as it requires a positive blood culture and rapid organism identification method, to determine eligibility for the tool and provide risk calculation before automated susceptibility testing, in order to appropriately benefit from these approaches [32,33]. Models have been developed elsewhere for general resistance to empiric therapy for both sepsis and bloodstream infections, and future diagnostic prediction model systematic reviews should evaluate the performance of available models for these clinical scenarios, to inform clinical practice [34,35]. As noted previously in the discussion, quality indicators for performance, including calibration and the clinical utility of models, were missing from the majority of studies, limiting the interpretability of the performance and useability of the models [36,37,38]. Additionally, the external validation studies were limited, further challenging the evaluation of model performance. Future studies should focus on the external validation of existing models and clinical utility determinations. Similarly, only 30% of the studies took place outside of the United States, which makes the generalizability or transportability of such approaches and related performance to non-US countries unclear [39]. Finally, our study was limited to only grey literature (e.g., conference proceedings) for two leading infectious disease conferences, which may have led to the omission of relevant research from other conferences, particularly outside of infectious diseases.

## 4. Materials and Methods

This systematic review and meta-analysis was reported in accordance with the Transparent Reporting of Multivariable Prediction Models for Individual Prognosis or Diagnosis: Checklist for Systematic Reviews and Meta-Analyses (TRIPOD-SRMA) (see Supplementary Table S2) [40]. This study was not preregistered.

### 4.1. Study Selection

We performed a literature search using PubMed and EMBASE, using the time period from each database’s inception to 3 September 2023, and including English-language studies. A search strategy for keywords related to Gram-negative AMR and prediction models was employed (Supplementary Table S3). In addition to searching these databases, we hand-searched infectious disease major conference proceedings (ECCMID and IDWeek) related to our topic for the last two years, covering the period of 2022–2023, to capture early-stage data that might still be pending full publication.

The eligible studies included cohort and case–control observational studies evaluating a population of adult patients (≥18 years old) with Enterobacterales BSI and risk prediction models, including risk scores. Both the derivation and validation model types were eligible for inclusion. The outcome of the risk-prediction models was AMR including ESBL and CRE. The timing for the model included models used at the suspected onset of bloodstream infection for both community-onset and healthcare-acquired infection. The setting of the models was for use in hospital-admitted or emergency department patients. Studies with multivariable models evaluating the association of predictors with AMR, but which did not attempt to develop the model for prediction (e.g., the predictive performance of the model was not evaluated), were excluded.

Two reviewers (M.F. and T.T.T.) independently assessed the identified records using the eligibility criteria above. Duplicates were initially removed, before articles were reviewed. A first-pass review of titles and abstracts was performed, with selection disagreements resolved through consensus. A second-pass review of full-text articles was performed, with the additional step of resolving final discordances though consensus. A bibliography review of the included articles (i.e., snowballing) was performed to identify additional articles for inclusion.

### 4.2. Data Extraction

Extracted data from the final selection of articles were included in the review into a standardized Microsoft Excel grid, based on guidance from the CHARMS checklist [41]. The PICO framework (population, index model, comparator model, outcome[s]) information was extracted for each study, using a standardized form [42]. Additionally, the source of the data, the sample size, the number of participants with AMR, the handling of missing data, the selection of predictors, the predictive performance (the overall model fit, discrimination, and calibration including standard errors or confidence intervals) and, where present, measures of clinical utility (e.g., net benefit, decision curve analysis) were reviewed. No authors were contacted for missing data.

### 4.3. Statistical Analysis

The study characteristics, including the model performance, were reported with descriptive statistics, or through figures. Meta-analysis using random effects modeling was performed for models where a specific model had been externally validated [43]. All the data analyses, including the meta-analyses, were performed using R version 4.0.1 and ‘metafor’ for analysis [44].

## 5. Conclusions

In conclusion, our review reveals that a multitude of resistance-in-Enterobacteriaceae bloodstream prediction models exist in the literature, with a minority of studies concerning the external validation of existing models. The models showed a moderate discrimination performance, with a lower performance in external validations. A local external validation of the performance of the model discrimination and calibration is needed in order to understand the use of these models in specific settings, along with potential clinical utility evaluations versus the baseline appropriate prescription in Enterobacteriaceae bacteremia. Due to the scarcity of reports on the interventional use of these models, further clinical evaluation is required, to determine their clinical utility and safety. Evaluating these or newer models will strengthen the assessment of the efficacy of such approaches and their impact on patient outcomes, thus informing healthcare institutions when it comes to potentially improving therapy management, reducing healthcare costs, and enhancing patient outcomes in Enterobacteriaceae bloodstream infections.

## Figures and Tables

**Table 1:** Study characteristics.

First Author, Year	Country	Design	Model Type	Sample Size	Resistance Type	Resistance Incidence
Andrews 2023 [9]	USA	Retrospective cohort	External validation of Lee 2017 and Augustine 2017	356	ESBL	11.5%
Augustine 2017 [10]	USA	Retrospective cohort	Derivation and internal validation	910	ESBL	4.6%
Cwengros 2020 [11]	USA	Retrospective cohort	External validation of Lee 2017 and Augustine 2017	451	ESBL	16%
Goodman 2016 [12]	USA	Retrospective cohort	Derivation and internal validation	1288	ESBL	15%
Goodman 2019 [13]	USA	Retrospective cohort	Derivation and internal validation	1288	ESBL	15%
Holmgren 2020 [14]	Sweden	Retrospective cohort	Derivation	625	ESBL	9%
Lee 2017 [15]	Taiwan	Retrospective cohort	Derivation	1141	ESBL	5.7%
Madrid-Morales 2021 [16]	USA	Retrospective cohort	External validation of Augustine, 2017; Goodman, 2016 and 2019; and Tumbarello, 2011	145	ESBL	13.8%
Tumbarello 2011 [17]	Italy	Retrospective case–control	Derivation and external validation	339; 510	ESBL	33.3%; 20%
Weston 2020 [18]	USA	Retrospective case–control	Derivation and internal validation	341	CRE	NA

**Table 2:** Model characteristics.

Study	Demographics	Hospitalization/Admission History	Medical History	Procedure/Intervention History	Medication Use History	Other
Augustine 2017 [10]	-	-	ESBL-E infection or colonization	GI/GU procedure	Number of prior BL/FQ courses	-
Goodman 2016 [12]	Age, ≥43 years	Hospitalization in ESBL high-burden region	ESBL-E infection or colonization, chronic indwelling vascular hardware	-	Days of antibiotic exposure	
Goodman 2019 [13]	-	Hospital care abroad, prior hospitalization	COPD, emphysema, ventilator dependence, indwelling hardware, MDRO colonization or infection	-	Weeks of antibiotic exposure	Source of infection
Hömgren 2019 [14]	-	Hospital care abroad	ESBL-E infection or colonization	-	-	-
Lee 2017 [15]	-	Prior hospitalization, recent ED visits	Urological diseases, diabetes mellitus	Invasive procedure	Antibiotic exposure	Nursing home residents
Tumbarello 2011 [17]	Age, ≥70 years	Recent hospitalization, admission from healthcare	Charlson comorbidity index, urinary catheterization	-	Previous therapy with β-lactams and/or fluoroquinolones	-
Weston 2020 [18]	-	Admitted >3 days	Prior CRE culture, liver disease, mechanical ventilation	-	Proton pump inhibitor, antibiotic exposure	Admission from SNF, no prior culture

NOTE. BL: β-lactams; COPD: chronic obstructive pulmonary disease; CRE: carbapenem-resistant Enterobacteriaceae; ED: emergency department; ESBL-E: extended-spectrum β-lactamase–producing Enterobacteriaceae; FQ: fluoroquinolones; GI: gastrointestinal; GU: genitourinary; MDRO: multidrug-resistant organisms; SNF: skilled nursing facility.

## Data Availability

The data used for all analyses and analytic code are available from the corresponding author upon reasonable request.

## Supplementary Materials

The following supporting information can be downloaded at: https://www.mdpi.com/article/10.3390/antibiotics12091452/s1, Table S1: Patient characteristics; Table S2: TRIPOD-SRMA Checklist for Reporting Systematic Reviews of Prediction Model Studies; Table S3: Keyword search.
