# Supplementary material for: Predicting Extended-Spectrum Beta-Lactamase and Carbapenem Resistance in Enterobacteriaceae Bacteremia: A Diagnostic Model Systematic Review and Meta-Analysis
Source: Antibiotics (Basel). 2023 Sep 17;12(9):1452. doi: 10.3390/antibiotics12091452 (PMC10525851; doi:10.3390/antibiotics12091452)
Supplement: Supplementary file 1 [file antibiotics-12-01452-s001.zip › antibiotics-2564687-supplementary.pdf]

Table S1. Patient Characteristics

Study	Age	Female	Race/Ethnicity	Comorbidities	Source of Infection	Organism
Andrews 2023 [9]	Median 62, IQR 47–72	54.8%	-	Diabetes 39.6%	-	*E. coli* 71.6%, *K. pneumoniae group* 18%, *K. oxytoca* 4.2%, *P. mirabilis* 4.0%, *Salmonella* spp. 2.2%
Augustine 2017 [10]	ESBL-E median 65, IQR 49–71<br><br>Non-ESBL-E median 66, IQR 55-78	55.9%	African American 49.7%, Other 3.3%, White 47%	Cancer 16.6%, Cirrhosis 4.8%, Diabetes 37%, ESRD 8.4%, Immunocompromised 11.3%	-	*E. coli* 66%, *K. pneumoniae* 23%, *K. oxytoca* 2%, *P. mirabilis* 9%, *Salmonella* spp. 1%
Cwengros 2020 [11]	Mean 65 ± SD 17	54%	African American 72.5%, Other 9.5%, White 18%	Cerebrovascular disease 12.9%, CHF 18.8%, COPD 17.5%, Dementia 12.6%, Diabetes 40.1%, ESRD 23.9%, Liver disease 12%, Solid tumor 18.8%; CCI mean 2 ± SD 2	-	*E. coli* 64%, *K. pneumoniae* 25%, *P. mirabilis* 11%
Goodman 2016 [12], 2019 [13]	ESBL-E mean 51, SD 18.4<br><br>Non-ESBL-E mean 56, SD 15.9	45.4%	African American 39.4%, Asian 4.9%, Latino 3.9%, Middle Eastern 3.9%, White 47.2%	CHF 7.5%, Dialysis 7.5%, HIV 4.5%, Immunosuppressant use 5.7%, Liver disease 7.2%, Solid organ transplant 13.5%, Structural lung disease 4.9%	Biliary 14.3%, BJI <1%, Catheter 15.5%, Intra-abdominal 23.8%, Pneumonia 5.7%, Skin 3.6%, Urinary 36.6%	*E. coli* 56%, *K. pneumoniae* 40%, *K. oxytoca* 4%
Holmgren 2019 [14]	ESBL-E median 72<br><br>Non-ESBL-E median 74	49.3%	-	Immunosuppressant use 16.8%	-	*E. coli* 77%, *Klebsiella* spp. 23%
Lee 2017 [15]	>65 yrs 63.4%	56.9%	-	Coronary artery disease 10.3%, Diabetes 39%, Hypertension 49.4%, Malignancy 26.9%, Neurological disorder 21%, Urological disease 6.7%	Biliary 12.1%, Intra-abdominal 11.7%, Liver abscess 5.5%, Pneumonia 9.1%, Primary bacteremia 6%, Urinary 52.4%	*E. coli* 72.4%, *K. pneumoniae* 24%, *P. mirabilis* 2.7%, and *K. oxytoca* 0.9%
Madrid Morales 2021 [16]	>70 yrs 45.0%	-	-	-	GI 24.1%, Multiple 3.2%, Pulmonary 5.5%, Skin 4.8%, Unknown 6.9%, Urinary 55.9%	*E. coli 75.2%*, *K. pneumoniae* 22.8%, *K. oxytoca* 1.4%, *K. aerogenes* <1%

Tumbarello 2011 [17]	Mean 65.9, SD 20.3	53.1%	-	AIDS 2.3%, Cerebrovascular disease 29.3%, Chronic obstructive pulmonary disease 16.7%, ESRD 21.4%, Diabetes mellitus 24.2%, CHF 50.2%, Hematological malignancy 7.4%, Liver disease 14%, Solid organ transplantation 4.2%, Solid tumor 29.3%; CCI ≥ 4: 45.1%	Biliary tract 4.7%, Blood 21.4%, Lower respiratory tract 8.8%, Skin and soft tissues 12.6%, Surgical wound 3.3%, Urinary 58.6%	Escherichia coli 77.2%, Klebsiella spp. 13.5%, Proteus mirabilis 10.2%
Weston 2020 [18]	CR-KP median 69, IQR 57-79<br><br>Non-CR-KP Median 68, IQR 56-77	47.5%	-	Chronic lung disease 26.9%, CHF 22.6%, Diabetes mellitus 37.5%, Hemiplegia or paraplegia 6.7%, Liver disease 23.2%, Malignancy 19.6%	Central line–associated 9.1%, Central nervous system 0.3%, Gastrointestinal 20.2%, Pneumonia 21.1%, Skin/soft tissue 4.4%, Unknown 23.7%, Urinary 31.7%	*Klebsiella pneumoniae* only

Table S2. TRIPOD-SRMA Checklist for reporting systematic reviews of prediction model studies

Section and topic	Item No	Checklist item	Page
**Title**			
Title	1	Identify the report as a systematic review or meta-analysis (or both) of diagnostic or prognostic model studies. Specify the target population and outcome(s) predicted as relevant to the review question.	1
**Abstract**			
Abstract	2	See the TRIPOD-SRMA Checklist for Abstracts	2
**Introduction**			
Rationale	3	Describe the rationale for the review in the context of existing knowledge.	1–2
Objectives	4	Provide an explicit statement of the objective(s) being addressed with reference to: target population, index and comparator models (as relevant), outcome(s), time (prediction horizon and intended moment of using the model), and setting.	2
**Methods**			
Study eligibility criteria	5	Specify study characteristics used as eligibility criteria, including any prediction models of specific interest, and whether development or validation studies (or both) were eligible.	7
Information sources	6	Specify all databases, registers, websites, organisations, reference lists and other sources searched or consulted to identify studies. Specify the date when each source was last searched or consulted.	7
Search strategy	7	Present the full search strategies for all databases, registers and websites, including any filters and limits used.	S1
Study selection process	8	Specify the methods used to decide whether a study met the inclusion criteria of the review, including how many reviewers screened each record and each report retrieved, whether they worked independently, and if applicable, details of automation tools used in the process.	7

Data collection process	9	Specify the methods used to collect data from study reports, including how many reviewers collected data from each report, whether they worked independently, any processes for obtaining or confirming data from study investigators, and if applicable, details of automation tools used in the process.	7
Data Items	10a	List and define all items for which data were sought from each study.	7
	10b	State the model performance measures that were sought (e.g., measures of calibration, discrimination, overall model fit, clinical utility).	7
	10c	Describe how any desired but unreported data items (items 10a, 10b) were handled (e.g., contacted authors, calculated from other reported information).	7
Risk of bias and applicability assessment	11	Specify the methods used to assess risk of bias in the included studies and their applicability to the review question. This should be done separately for each model development and validation. Include details of any tool(s) used, how many reviewers assessed each study and whether they worked independently.	-
Synthesis methods	12a	Describe any methods for synthesising estimates of performance measures for each model. If meta-analysis was carried out, describe the methods used, including any transformations of data prior to pooling, how any heterogeneity in model performance was quantified and handled, and software package(s) used.	-
	12b	Describe any methods used to explore possible causes of heterogeneity in model performance (e.g., subgroup analysis, meta-regression), including whether or not they were planned.	-

**Section and topic**	**Item No**	**Checklist item**	**Page**
	12c	Describe any sensitivity analyses conducted to assess robustness of the synthesised results.	-
Certainty assessment	13	Describe any methods used to assess certainty (or confidence) in the body of evidence for a prediction model.	-
**Results**			
Study selection	14	Describe the results of the search and selection process, from the number of records identified in the search to the number of studies and models included in the review, ideally using a flow diagram.	2
Study and model characteristics	15	Present study characteristics and model details extracted (as per Item 10a), and cite the study reports.	2
Risk of bias and applicability	16	Present results of risk of bias and applicability assessment. This should be done separately for each model development and validation in each included study.	-
Results of model performance in individual studies	17	Present performance estimates and confidence intervals for each model and all evaluations, including whether they relate to the internal or external validation performance. If internal, give details of the method.	3–5
Results of syntheses	18a	Present the results of any synthesis of model performance, together with details of which study estimates contributed. If meta-analysis was carried out, then for each model and performance measure, present summary results, confidence/credible intervals and measures of heterogeneity. Forest plots may be useful.	-
	18b	For each model, present results of all investigations of possible causes of heterogeneity in model performance.	-
	18c	Present results of all sensitivity analyses conducted to assess the robustness of the synthesised results.	-

Certainty of evidence	19	Present any assessments of certainty (or confidence) in the body of evidence for each prediction model of interest.	-
**Discussion**			
Summary of evidence	20	Summarise the main findings including the strengths and limitations of the evidence.	6
Limitations	21	Discuss the strengths and limitations of the review process.	
Implications	22	Discuss implications of the results in the context of other evidence and for practice, policy, and future research.	
**Other information**			
Registration and protocol	23a	Provide registration information for the review, including register name and registration number, or state that the review was not registered.	8
	23b	Indicate where the review protocol can be accessed, or state that a protocol was not prepared.	-
	23c	Describe and explain any amendments to information provided at registration or in the protocol.	-
Support	24	Describe sources of financial or non-financial support for the review, and the role of the funders or sponsors in the review.	8

**Section and topic**	**Item No**	**Checklist item**	**Page**
Competing interests	25	Declare any competing interests of review authors.	8
Availability of data, code, and other materials	26	Report which of the following are publicly available and where they can be found: template data collection forms; data extracted from included studies; data used for all analyses; analytic code; any other materials used in the review.	8

This checklist appears in appendix 2 of Snell KIE, Levis B, Damen JAA, et al. Transparent reporting of multivariable prediction models for individual prognosis or diagnosis: checklist for systematic reviews and meta-analyses (TRIPOD-SRMA). *BMJ* 2023;381:e073538. doi:10.1136/bmj-2022-073538.

Table S3: Keyword search

**Query**
((ESBL OR ESBL-E OR extended-spectrum beta-lactamase OR CRE OR carbapenem resistant Enterobacteriaceae) OR (ESBL risk factors OR CRE risk factors)) OR ("gram-negative bacterial infections") AND (("antibiotic resistance" OR "antimicrobial resistance") OR (multi-drug resistance)) AND ((scoring models OR "risk factors") OR (prediction model OR algorithm OR predictive models))
